# Andexanet Alfa for Edoxaban Reversal and Associated Thromboelastography Changes in Surgical Pulmonary Embolectomy

**DOI:** 10.7759/cureus.62370

**Published:** 2024-06-14

**Authors:** Yu Fukuda, Koichi Yoshinaga, Shin Kondo, Yusuke Iizuka, Masamitsu Sanui

**Affiliations:** 1 Department of Anesthesiology and Critical Care Medicine, Jichi Medical University Saitama Medical Center, Saitama, JPN; 2 Department of Anesthesiology and Critical Care Medicine, Jichi Medical University, Saitama, JPN

**Keywords:** venoarterial extracorporeal membrane oxygenation, thromboelastography, factor xa inhibitor, edoxaban, citrated kaolin-reaction time, cardiopulmonary bypass, cardiac surgery, andexanet alfa

## Abstract

Andexanet alfa neutralizes factor Xa inhibitors in critical bleeding situations. However, in cardiac surgery with cardiopulmonary bypass (CPB), heparin resistance induced by andexanet alfa should be a concern, and the lack of point-of-care monitoring of plasma concentration of factor Xa inhibitors makes it difficult to decide when to administer andexanet alfa.

A 69-year-old man underwent emergency surgery for acute pulmonary thromboembolism. The patient had been on edoxaban until the day before the surgery. Withdrawal from CPB required venoarterial extracorporeal membrane oxygenation due to right heart failure, followed by severe bleeding that required massive transfusion. Despite adequate coagulation factor replacement, bleeding persisted and citrated kaolin-reaction time (CK-R) on thromboelastography (TEG) was prolonged. Administering andexanet alfa achieved excellent hemostasis without any thrombosis and normalized the prolonged CK-R of TEG.

This is the first report of a change in TEG findings before and after administration of andexanet alfa in a cardiac surgery patient taking factor Xa inhibitor. Monitoring CK-R in TEG may help evaluate the anticoagulant effect of factor Xa inhibitors and the reversal effect of andexanet alfa.

## Introduction

Direct oral anticoagulants (DOACs) are widely used for the prevention and treatment of thromboembolism due to their advantages over vitamin K antagonists, including fewer monitoring requirements, rapid onset of action, and minimal drug and food interactions [1]. However, DOACs are associated with the risk of acute major bleeding, and, in 2018, the U.S. Food and Drug Administration approved andexanet alfa as an antidote to reverse the effects of factor Xa inhibitors.

Andexanet alfa is a modified, recombinant form of the factor Xa protein that has been developed as an antidote for factor Xa inhibitors [2]. Although andexanet alfa is catalytically inactive and lacks the ability to convert prothrombin to thrombin, it retains the ability to bind direct factor Xa inhibitors. It counteracts the effects of direct factor Xa inhibitors by acting as a decoy protein. The ANNEXA-4 study, the first clinical trial evaluating the efficacy of andexanet alfa, focused on patients with acute major bleeding who had received factor Xa inhibitors within the previous 18 hours [3]. It was shown that the activity of the factor Xa inhibitor was reduced and good hemostasis was achieved 12 hours after administration of andexanet alfa; however, patients scheduled for surgery were excluded from the study.

Two primary concerns arise when administering andexanet alfa to patients undergoing cardiac surgery. First, andexanet alfa may induce heparin resistance, complicating anticoagulation management in patients requiring cardiopulmonary bypass (CPB) or extracorporeal membrane oxygenation (ECMO) [4]. Second, the lack of rapid factor Xa inhibitor monitoring tests in clinical settings means that whether or not andexanet alfa is indicated for a particular patient depends solely on documentation of the patient’s last oral dose.

Here, we present a patient on edoxaban who underwent emergency surgical embolectomy for acute pulmonary embolism and developed severe bleeding during postoperative venoarterial ECMO management. Despite adequate coagulation factor replacement, the bleeding continued, and thromboelastography (TEG) showed a prolonged citrated kaolin-reaction time (CK-R). Consequently, we determined the effect of DOAC to be prolonged and administered andexanet alfa, which resulted in good hemostasis and a shortened CK-R value. TEG was extremely useful in detecting the bleeding tendency associated with prolonged factor Xa inhibitor effects and confirming the neutralizing effect of andexanet alfa in a point-of-care setting.

## Case presentation

A 69-year-old male with a history of asthma, hyperlipidemia, hyperuricemia, and subarachnoid hemorrhage was diagnosed with deep vein thrombosis and acute pulmonary embolism. He was on edoxaban 60 mg daily until the day before his transfer to our hospital. On the day of the transfer, he suddenly went into cardiac arrest, and venoarterial ECMO was introduced. The patient was transferred to our hospital for emergent surgery for acute pulmonary thromboembolism.

On arrival at our hospital, he was already intubated and sedated with midazolam and fentanyl. An arterial cannula was inserted into the right femoral artery, and a venous cannula into the left femoral vein, with an ECMO flow rate of around 3 L/minute. The mean arterial pressure was around 80 mmHg without pulse pressure. The blood tests revealed a hemoglobin level of 11.7 g/dL, platelet count of 3.8 × 10^9^/L, prothrombin time-international normalized ratio (PT-INR) of 6.98, activated partial thromboplastin time (APTT) of 34.8 seconds, and fibrinogen level of 180 mg/dL. Aspartate transferase (AST) was 2,643 U/L, alanine transferase (ALT) was 1,219 U/L, lactate dehydrogenase (LDH) was 2,981 U/L, total bilirubin was 1.99 mg/dL, and serum creatinine was 2.22 mg/dL. The patient exhibited persistent bleeding at the site of the ECMO cannula insertion due to coagulation abnormalities, and after receiving eight units of fresh frozen plasma and two units of apheresis platelet concentrates, he was admitted to the operating room.

Following median sternotomy, an unfractionated heparin bolus of 300 units/kg (20,000 units) was administered, and the activated clotting time (ACT) was prolonged to 862 seconds (control ACT was 283 seconds). Subsequently, CPB was established (arterial cannula: ascending aorta, venous cannulae: superior/inferior vena cava). Thrombi obstructing the main trunk of the bilateral pulmonary arteries were identified and removed. The CPB time was 106 minutes, the aortic cross-clamp time was 46 minutes, and the minimum nasopharyngeal temperature during CPB was 29.8°C; circulatory arrest was not required. No additional heparin administration was required during CPB; the last ACT before withdrawal from CPB was 764 seconds. Intraoperative transesophageal echocardiography during weaning from CPB showed the right ventricular function was severely impaired (tricuspid annular plane systolic excursion: 15 mm), so the decision was made to continue with venoarterial ECMO owing to the difficulty in weaning from CPB. The ACT failed to normalize after protamine administration (200 mg), remaining at 343 seconds, and diffuse microvascular bleeding was observed throughout the operative field. To achieve hemostasis, we administered six units of packed red blood cells, 14 units of fresh frozen plasma, four units of apheresis platelet concentrates, six units of cryoprecipitate, and 2 g of fibrinogen concentrate. Subsequent blood tests revealed a platelet count of 10.3 × 10^9^/L, PT-INR of 2.50, APTT of 25.7 seconds, fibrinogen level of 262 mg/dL, and thromboelastography (TEG 6s, Haemonetics Corporation, Boston, MA, USA) revealed a prolonged CK-R of 14.3 minutes and citrated kaolin with heparinase-reaction time (CKH-R) of 13.2 minutes (Table 1). Although the results of TEG suggested residual effects of edoxaban, we decided not to administer andexanet alfa as the bleeding had decreased in the operative field and a potential thrombotic complication in ECMO was a concern. The surgery was completed; intraoperative blood loss was 1,150 mL.

**Table 1:** Serial change in thromboelastography parameters before and after andexanet alfa administration. Reference range: CK-R, 4.6-9.1 minutes; CKH-R, 4.3-8.3 minutes; CK-MA, 52-69 mm; CFF-MA, 15-32 mm. AA = andexanet alfa; CPB = cardiopulmonary bypass; CK-R = citrated kaolin-reaction time; CKH-R = citrated kaolin heparinase-reaction time; CK-MA = citrated kaolin-maximal amplitude; CFF-MA = citrated functional fibrinogen-maximal amplitude

	Before CPB	Intraoperative (before chest closure)	Postoperative (before AA administration)	After AA administration
CK-R (minutes)	26.0	14.3	19.9	7.3
CKH-R (minutes)	10.1	13.2	18.8	7.5
CK-MA (mm)	49.6	56.4	48.8	53.1
CFF-MA (mm)	20.8	20.8	23.7	20.0

**Figure 1:**
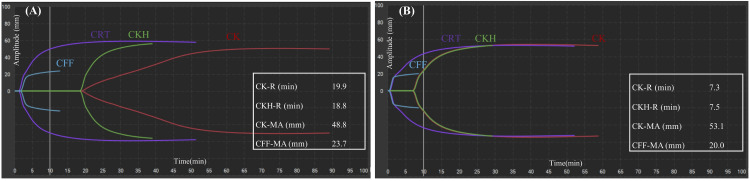
(A) Thromboelastography waveform before andexanet alfa administration. (B) Thromboelastography waveform after andexanet alfa administration. The vertical axis represents amplitude (mm) and the horizontal axis represents clotting time (minutes). Reference range: CK-R, 4.6-9.1 minutes; CKH-R, 4.3-8.3 minutes; CK-MA, 52-69 mm; CFF-MA, 15-32 mm. CK-R = citrated kaolin-reaction time; CKH-R = citrated kaolin heparinase-reaction time; CK-MA = citrated kaolin-maximal amplitude; CFF-MA = citrated functional fibrinogen-maximal amplitude

After admission to the intensive care unit (ICU), substantial bleeding from the mediastinal chest tube persisted (300-400 mL/hour), and surgical re-exploration was considered. We administered two units of packed red blood cells, two units of fresh frozen plasma, and one unit of apheresis platelet concentrates. Blood tests (three hours post-ICU admission) revealed a platelet count of 9.3 × 10^9^/L, PT-INR of 2.53, APTT of 28.7 seconds, fibrinogen level of 241 mg/dL, and TEG showed a prolonged CK-R of 19.9 minutes and CKH-R of 18.8 minutes (Table 1, Figure 1, Panel A).

Considering the possibility of residual edoxaban effect, andexanet alfa (400 mg bolus over 30 minutes followed by 4 mg/minute infusion over two hours) was administered as a last resort. Immediately after bolus administration, the chest tube output substantially decreased. TEG revealed a normalized CK-R of 7.3 minutes and CKH-R of 7.5 minutes immediately after andexanet alfa administration (Table 1, Figure 1, Panel B).

Continuous infusion of heparin was started after 10 hours of administration of andexanet alfa to ensure that hemostasis was achieved. The patient was managed on venoarterial ECMO without thrombotic complications, weaned from ECMO on postoperative day seven, and transferred to the previous hospital for rehabilitation on day 14 with full recovery of consciousness. Written informed consent was obtained from the patient for publication of this case report and any accompanying images.

## Discussion

We describe a case of fatal bleeding after emergency surgery for acute pulmonary thromboembolism in a patient taking edoxaban. Administration of andexanet alfa, along with the transfusion of sufficient amounts of blood products, resulted in excellent hemostasis and normalization of the reaction time of TEG, which had been abnormally prolonged before administration. To our knowledge, this is the first report of a change in TEG findings before and after administration of andexanet alfa in patients undergoing cardiac surgery with CPB.

No clinical trials have reported the efficacy of andexanet alfa in cardiac surgery with CPB, and its clinical use is based on the experience reported in several case reports [4-7]. These reports often describe heparin resistance following administration of andexanet alfa before CPB, as andexanet alfa also binds to the heparin-antithrombin complex and neutralizes its anticoagulant effect [2]. Therefore, the administration of andexanet alfa following CPB withdrawal may be a reasonable alternative. In fact, one case report has documented good hemostasis and no thromboembolism following andexanet alfa administration after CPB withdrawal, leading to our decision to follow this approach [8]. However, given that venoarterial ECMO was still required postoperatively, there was a significant concern about the subsequent risk of thrombus formation in the ECMO circuit due to the altered heparin response induced by andexanet alfa. Therefore, we first attempted to achieve hemostasis without andexanet alfa, but the hemorrhage remained uncontrollable. Andexanet alfa was administered as a last-resort intervention after ICU admission. As a result, dramatic hemostasis was achieved, and the subsequent ECMO management progressed without any thromboembolic events. If an intracircuit thrombus had formed under the influence of andexanet alfa, we might have considered using antithrombin, as there are case reports recommending its use in conjunction with treatments for heparin resistance [5]. In addition, depending on the extent of the intracircuit thrombus, circuit replacement might have been necessary.

The patient’s marked bleeding tendency may have been associated with the following factors: the prolonged edoxaban effect, decreased coagulation factor activity due to CPB and hemodilution, heparin rebound, and hepatic and renal impairment due to cardiac arrest and CPB. After withdrawal of CPB, we adequately supplemented coagulation factors, fibrinogen, and platelets, and blood tests before administration of andexanet alfa confirmed that platelet counts, APTT, and fibrinogen levels were satisfactory (only PT was prolonged). TEG indicated satisfactory recovery of fibrinogen and platelet function, as the maximal amplitude of CK and citrated functional fibrinogen were in the adequate range (Table 1). The reaction time of the CK/CKH assay showed no significant difference, suggesting that heparin rebound was an unlikely cause. Therefore, we administered andexanet alfa, which resulted in immediate hemostasis. Moreover, a marked improvement of the CK-R and CKH-R was achieved immediately after andexanet alfa administration (Table 1). Although the plasma concentration of edoxaban was unknown, the clinical course suggested that the bleeding was caused by the prolonged edoxaban effect. In this patient, edoxaban was taken 44 hours before andexanet alfa administration. Edoxaban has a half-life of 10-14 hours, and renal clearance accounts for approximately 50% of its total clearance, while hepatic metabolism and excretion account for the remaining 50% [9]. The patient had significant hepatic and renal dysfunction on admission (AST: 2,643 U/L, ALT: 1,219 U/L, LDH: 2,981 U/L, total bilirubin: 1.99 mg/dL, serum creatinine: 2.22 mg/dL), which may have prolonged the effect of edoxaban.

Point-of-care coagulation monitoring with TEG is recommended to reduce bleeding and transfusion in cardiac surgery patients [10]. TEG may be beneficial not only for diagnosing the cause of bleeding tendency but also for confirming the effect of factor Xa inhibitors in cases when the plasma concentration of factor Xa inhibitor is unknown but bleeding tendency persists, and for confirming the neutralizing effect of andexanet alfa. Traditional methods for measuring the plasma concentration of factor Xa inhibitors, such as liquid chromatography-mass spectrometry and drug-calibrated chromogenic anti-Xa assay [11], are challenging and impractical during emergency surgery. Research comparing TEG 6s parameters between healthy individuals and those taking factor Xa inhibitors found a significant prolongation of CK-R in the latter [12]. In another study, rivaroxaban administration significantly prolonged CK-R with increased plasma concentration, whereas apixaban did not [13]. Furthermore, in vitro evaluations using TEG 5000 with blood samples from healthy volunteers spiked with factor Xa inhibitors and/or andexanet alfa revealed that andexanet alfa could normalize the prolonged reaction time caused by factor Xa inhibitors [14]. Although the TEG anti-factor Xa assay would allow more sensitive detection of factor Xa inhibitors [12,13], they are not currently available in clinical practice. Monitoring CK-R with TEG 6s may be valuable in determining the prolonged effect of factor Xa inhibitors and confirming the neutralizing effect of andexanet alfa in a point-of-care setting.

## Conclusions

In summary, we experienced life-threatening bleeding after CPB in a patient taking edoxaban, who underwent emergency surgical embolectomy for acute pulmonary thromboembolism. Administration of andexanet alfa in patients requiring ECMO carries a risk of fatality due to thrombotic complications, but as a last resort, andexanet alfa was administered for bleeding tendency, resulting in excellent hemostasis and normalization of TEG reaction time. Monitoring CK-R in TEG may help determine the prolonged effect of factor Xa inhibitors and the reversal efficacy of andexanet alfa.
